# MicroRNA-26b suppresses the NF-κB signaling and enhances the chemosensitivity of hepatocellular carcinoma cells by targeting TAK1 and TAB3

**DOI:** 10.1186/1476-4598-13-35

**Published:** 2014-02-24

**Authors:** Na Zhao, Ruizhi Wang, Liangji Zhou, Ying Zhu, Jiao Gong, Shi-Mei Zhuang

**Affiliations:** 1 Key Laboratory of Gene Engineering of the Ministry of Education, State Key Laboratory of Biocontrol, School of Life Sciences, Sun Yat-sen University, Xin Gang Xi Road 135#, Guangzhou 510275, P R China; 2 Key Laboratory of Liver Disease of Guangdong Province, The Third Affiliated Hospital, Sun Yat-sen University, Guangzhou 510630, P R China

**Keywords:** miR-26b, Noncoding RNA, NF-κB signaling, Hepatocellular carcinoma, TAK1, TAB3

## Abstract

**Background:**

Abnormal activation of the NF-κB pathway is closely related to tumorigenesis and chemoresistance. Therefore, microRNAs that possess the NF-κB inhibitory activity may provide novel targets for anti-cancer therapy. miR-26 family members have been shown to be frequently downregulated in hepatocellular carcinoma (HCC) and correlated with the poor survival of HCC patients. To date, there is no report disclosing the regulatory role of miR-26 on the NF-κB pathway and its biological significance.

**Methods:**

The effects of miR-26b on the NF-κB signaling pathway and the chemosensitivity of cancer cells were examined in two HCC cell lines, QGY-7703 and MHCC-97H, using both gain- and loss-of-function studies. The correlation between miR-26b level and apoptosis rate was further investigated in clinical HCC specimens.

**Results:**

Both TNFα and doxorubicin treatment activated the NF-κB signaling pathway in HCC cells. However, the restoration of miR-26b expression significantly inhibited the phosphorylation of IκBα and p65, blocked the nuclear translocation of NF-κB, reduced the NF-κB reporter activity, and consequently abrogated the expression of NF-κB target genes in TNFα or doxorubicin-treated HCC cells. Furthermore, the ectopic expression of miR-26b dramatically sensitized HCC cells to the doxorubicin-induced apoptosis, whereas the antagonism of miR-26b attenuated cell apoptosis. Consistently, the miR-26b level was positively correlated with the apoptosis rate in HCC tissues. Subsequent investigations revealed that miR-26b inhibited the expression of TAK1 and TAB3, two positive regulators of NF-κB pathway, by binding to their 3’-untranslated region. Moreover, knockdown of *TAK1* or *TAB3* phenocopied the effects of miR-26b overexpression.

**Conclusions:**

These data suggest that miR-26b suppresses NF-κB signaling and thereby sensitized HCC cells to the doxorubicin-induced apoptosis by inhibiting the expression of TAK1 and TAB3. Our findings highlight miR-26b as a potent inhibitor of the NF-κB pathway and an attractive target for cancer treatment.

## Background

Nuclear factor κB (NF-κB) is a family of transcription factors that are implicated in many physiological and pathological processes, including immunity, inflammation, carcinogenesis and chemoresistance [1,2]. In mammals, the NF-κB family consists of five structurally related proteins, RelA (p65), NF-κB-1 (p105/p50), NF-κB-2 (p100/p52), RelB, and c-Rel, which form various homodimers and heterodimers. The predominant form of NF-κB consists of p50 and p65 subunits. In most cell types, NF-κB is mainly trapped in the cytoplasm in an inactive form bound to IκB proteins, the inhibitors of NF-κB. In response to stimuli like tumor necrosis factor alpha (TNFα) or interleukin-1β (IL1β), TGFβ-activated kinase-1 (TAK1) and its adaptors TAB2/3 are recruited to the receptor proximal signaling complex, leading to the activation of IκB kinase (IKK). The activated IKK phosphorylates IκB proteins and triggers the ubiquitination and degradation of IκB, which allows p50/p65 heterodimer to be released, translocate to the nucleus and act as a sequence-specific DNA-binding transcription factor. Meanwhile, IKK phosphorylates the Ser536 of p65 and thereby enhances the transactivation activity of NF-κB [1,3]. In addition to extracellular ligands that signal through membrane receptors, the chemotherapy-induced DNA damage also activates NF-κB in some cell contexts [1,4]. Many potent anti-apoptosis genes are transactivated by NF-κB [1,2]. Therefore, the activation of NF-κB may desensitize cells to apoptosis and thereby promote cancer progression. Unfortunately, abnormal activation of the NF-κB pathway is a common phenomenon in cancer cells [2].

MicroRNAs (miRNAs) are evolutionarily conserved small non-coding RNAs that suppress protein expression by binding to the 3’-untranslated region (3’UTR) of target mRNA. Several miRNAs have been reported to modify cell behavior by regulating the NF-κB pathway. For example, miR-301a, miR-30e* and miR-182 promote NF-κB activity and thereby enhance tumor growth, invasiveness or angiogenesis [5-7]. On the contrary, miR-15/16/195 and miR-146a/b have been shown to impair NF-κB activity, thus reducing the proliferation and metastasis of tumor cells [8-10]. Very few miRNAs have been characterized to affect chemosensitivity by regulating the NF-κB pathway: miR-143 sensitizes colorectal cancer cells to 5-fluorouracil treatment by downregulating ERK5, Bcl-2 and p65 expression [11]; miR-146a enhances the chemosensitivity of NK/T cell lymphoma to etoposide by targeting TRAF6 [12]. Clearly, identification of miRNAs that target NF-κB signaling may provide novel molecular targets for cancer therapy.

It is reported that NF-κB signaling is frequently activated in hepatocellular carcinoma (HCC) [2,13]. In order to uncover the HCC-associated miRNAs which may regulate the NF-κB pathway, we predicted the targets of those deregulated miRNAs that we found in HCC tissues [14], using target prediction algorithms (TargetScan). Among the miRNAs that were predicted to target the regulators of NF-κB pathway, miR-26b stood out as a potential candidate, with TAK1 and TAB3 as its putative targets. Studies from us and other groups show that the miR-26 family (miR-26a/b) is frequently downregulated in multiple types of cancer, including HCC, breast cancer, nasopharyngeal carcinoma and melanoma [15-19]. To date, there is no report disclosing the regulatory role of miR-26a/b on the NF-κB pathway and its biological significance. Because of the share of common seed sequences for target recognition, members of a miRNA family usually play similar, if not identical, roles. Therefore, we explored the impact of miR-26b on NF-κB signaling and its biological significance. We found that miR-26b suppressed the TNFα- and doxorubicin-activated NF-κB signaling in HCC cells, and sensitized cancer cells to the doxorubicin-induced apoptosis by targeting TAK1 and TAB3.

## Results

### miR-26b suppresses the TNFα-induced NF-κB signaling in HCC cells

**Figure 1:**
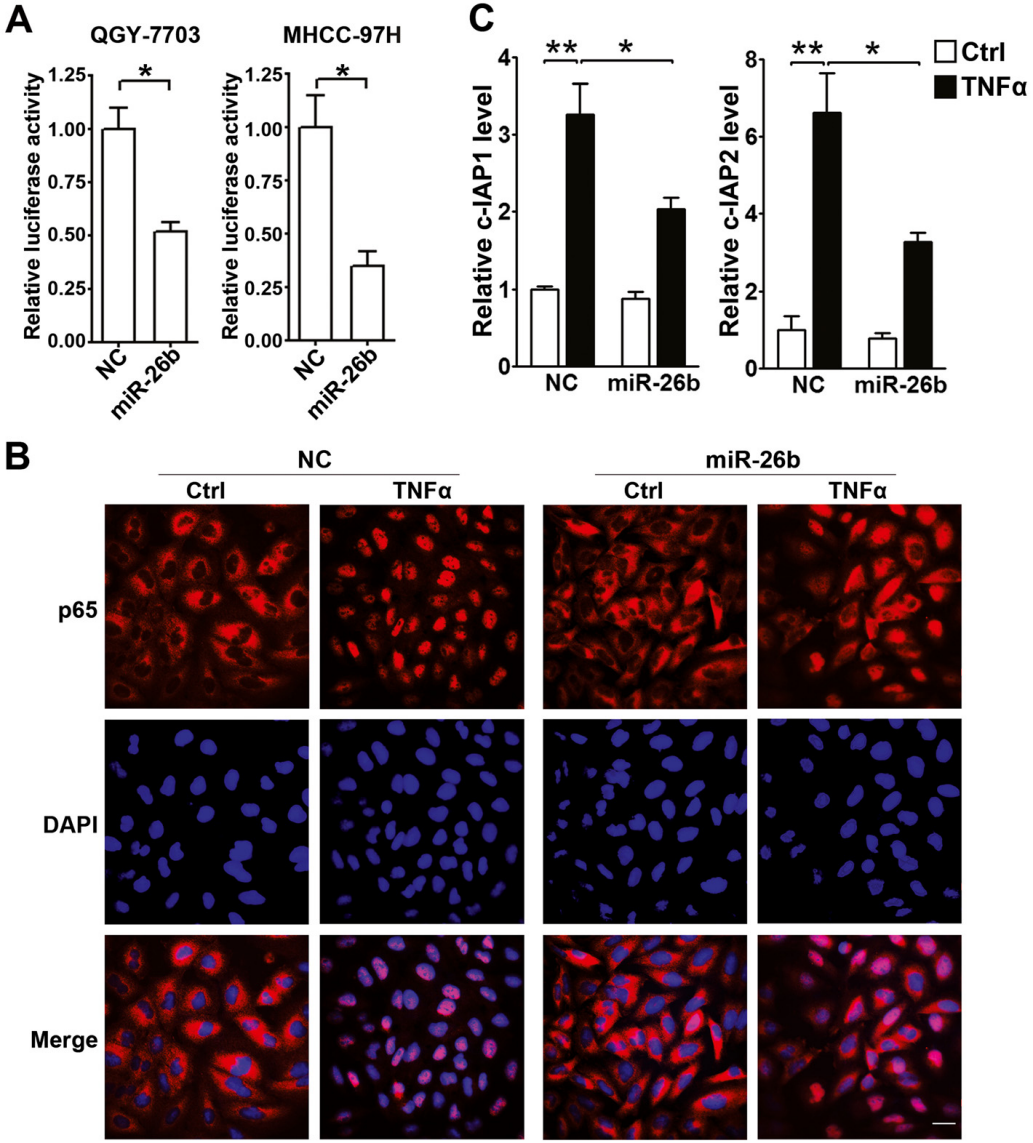
**miR-26b suppresses the TNFα-stimulated NF-κB signaling. (A)** miR-26b inhibited the TNFα-induced NF-κB reporter activity. HCC cells were first transfected with NC or miR-26b duplexes, followed by co-transfection of pRL-TK and pNF-κB-Luc or pTAL-Luc, treatment with TNFα, and analysis for luciferase activity. **(B)** miR-26b blocked the TNFα-induced nuclear translocation of NF-κB. QGY-7703 cells transfected with NC or miR-26b were untreated (Ctrl) or treated with TNFα before immunofluorescent staining for p65 (red). The nuclei were stained blue with DAPI. Scale bar, 10 μm. **(C)** miR-26b suppressed the TNFα-induced expression of NF-κB target genes. QGY-7703 cells transfected with NC or miR-26b were treated with 20 ng/ml TNFα for 3 hours before qPCR analysis. *, *P* < 0.05; **, *P* < 0.01.

To explore the impact of miR-26b on NF-κB signaling, miR-26b or its negative control (NC) duplex was co-transfected with the luciferase reporter that contained multiple NF-κB binding sites in its promoter. Compared with the NC-transfectants, miR-26b-transfected QGY-7703 and MHCC-97H cells displayed a significantly lower NF-κB reporter activity in response to TNFα, the classical NF-κB activator (Figure 1A).

It has been demonstrated that the activated NF-κB will translocate into nucleus and induce the transcriptional activation of anti-apoptosis genes, like *c-IAP1* and *c-IAP2*[20]. Consistently, we observed a prominent nuclear translocation of NF-κB in the TNFα-treated NC-transfectants. However, transfection with miR-26b efficiently blocked the TNFα-induced NF-κB nuclear translocation in both QGY-7703 (Figure 1B) and MHCC-97H cells (Additional file 1: Figure S1A). Furthermore, TNFα stimulation upregulated the mRNA levels of *c-IAP1* and *c-IAP2* in the NC-transfectants, but this effect was significantly abrogated by the transfection of miR-26b (Figure 1C and Additional file 1: Figure S1B).

**Figure 2:**
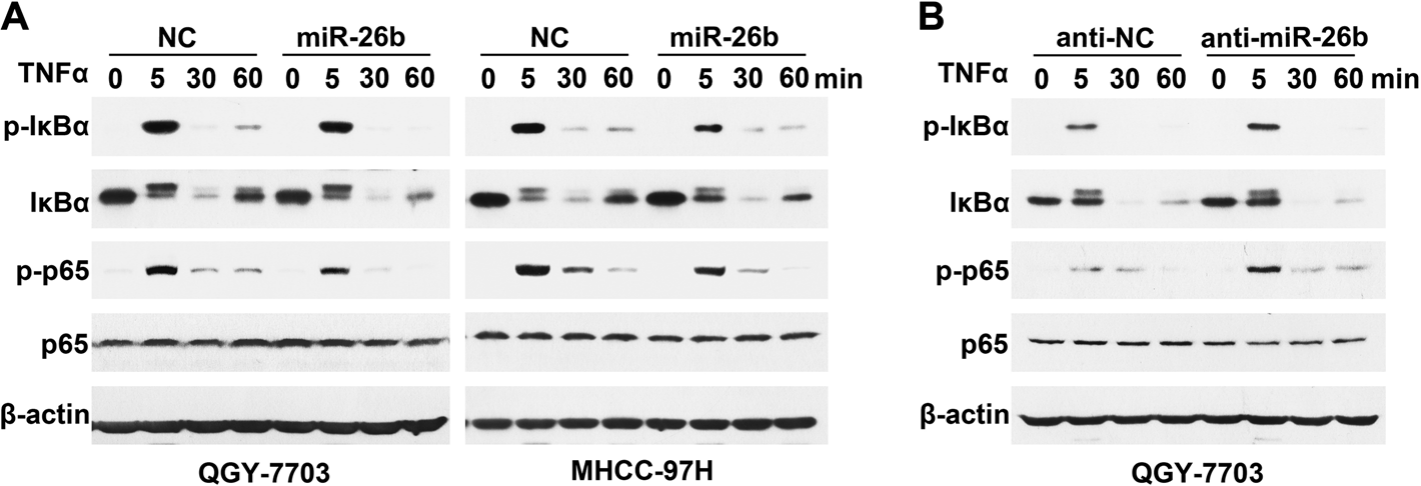
**miR-26b inhibits the TNFα-induced phosphorylation of IκBα and p65. (A)** Introduction of miR-26b attenuated the TNFα-induced phosphorylation of IκBα and p65. HCC cells transfected with NC or miR-26b duplexes were treated with 20 ng/ml TNFα for the indicated time before immunoblotting. **(B)** Antagonism of miR-26b enhanced the TNFα-induced phosphorylation of IκBα and p65. QGY-7703 cells transfected with anti-NC or anti-miR-26b were treated with 2 ng/ml TNFα for the indicated time.

Next, the influence of miR-26b on the signaling molecules of NF-κB pathway was investigated. As reported, TNFα treatment significantly increased the phosphorylation of IκBα and p65 in control cells (Figure 2A), suggesting the activation of NF-κB signaling. Notably, the TNFα-induced phosphorylation of IκBα and p65 was much less evident in the miR-26b-transfectants, compared with the control cells (Figure 2A). In contrast, the antagonism of endogenous miR-26b by anti-miR-26b (Additional file 2: Figure S2) enhanced the TNFα-stimulated NF-κB signaling (Figure 2B).

Collectively, these data indicate that miR-26b may suppress NF-κB signaling by attenuating the phosphorylation of IκBα and p65.

### TAK1 and TAB3 are direct targets of miR-26b

As mentioned above, TAK1 and TAB3 are the upstream positive regulators of the NF-κB pathway [3] and their 3’UTRs contain putative miR-26b-binding sites (Additional file 3: Figure S3), as predicted by TargetScan (Release 5.2, http://www.targetscan.org/vert_50/, in which *TAK1* and *TAB3* are designated as *MAP3K7* and *MAP3K7IP3*, respectively). We therefore examined whether TAK1 and TAB3 were direct targets of miR-26b which mediated the suppressive effect of miR-26b on NF-κB signaling.

**Figure 3:**
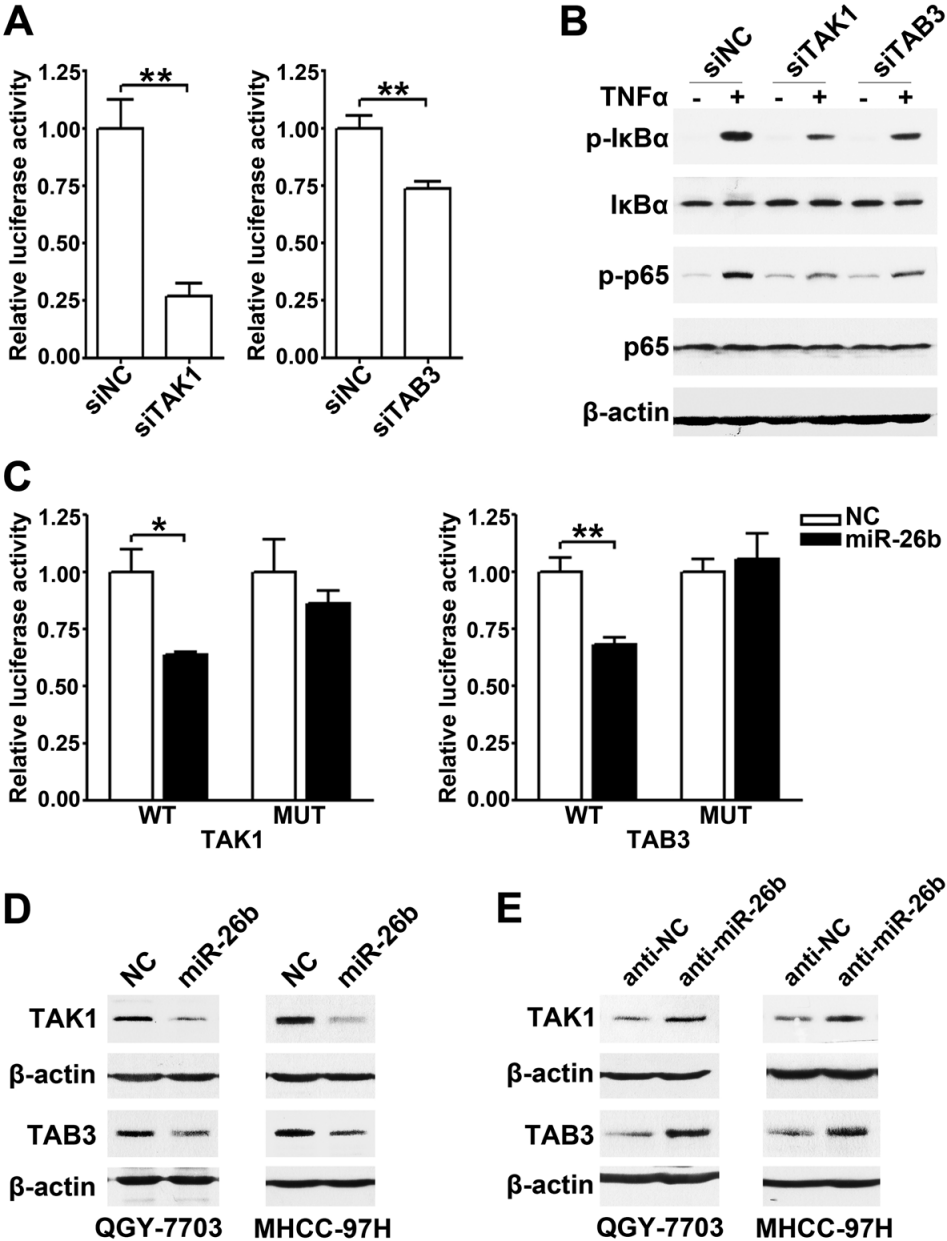
**miR-26b suppresses NF-κB signaling by targeting TAK1 and TAB3. (A)** Knockdown of *TAK1* or *TAB3* inhibited the TNFα-induced NF-κB reporter activity. QGY-7703 cells were treated and analyzed as in Figure 1A. **(B)** Knockdown of *TAK1* or *TAB3* attenuated the TNFα-induced phosphorylation of IκBα and p65. QGY-7703 cells transfected with siNC (*lanes* 1, 2), siTAK1 (*lanes* 3, 4) or siTAB3 (*lanes* 5, 6) were untreated (-) or treated with 20 ng/ml TNFα (+) for 3 minutes before immunoblotting. **(C)** miR-26b repressed the activity of the luciferase reporter containing the wild-type 3’UTR of *TAK1* or *TAB3*. QGY-7703 cells were co-transfected with NC or miR-26b duplexes, pRL-TK and a firefly luciferase reporter plasmid carrying the wild-type (WT) or the mutant (MUT) 3’UTR of *TAK1* or *TAB3* before luciferase activity analysis. **(D)** Expression of miR-26b reduced the protein levels of cellular TAK1 and TAB3. HCC cells were transfected with NC or miR-26b duplexes for 48 hours before immunoblotting. **(E)** Antagonism of endogenous miR-26b enhanced the levels of TAK1 and TAB3 proteins. HCC cells were transfected with anti-NC or anti-miR-26b for 48 hours before immunoblotting. *, *P* < 0.05; **, *P* < 0.01.

As shown, knockdown of either *TAK1* or *TAB3* gene by small interfering RNA (siRNA) (Additional file 4: Figure S4A and B) abated the TNFα-induced activity of NF-κB reporter (Figure 3A) and phosphorylation of IκBα and p65 (Figure 3B), which mimicked the effect of miR-26b overexpression in the same cell models. Furthermore, dual-luciferase reporter analysis showed that the co-expression of miR-26b significantly inhibited the activity of firefly luciferase that carried the wild-type but not mutant 3’UTR of *TAK1* or *TAB3* (Figure 3C), indicating that miR-26b may suppress gene expression through its binding sequences at the 3’UTR of *TAK1* and *TAB3*. Next, the effect of miR-26b on the endogenous cellular expression of its potential targets was examined. The results revealed that the introduction of miR-26b diminished the expression of TAK1 and TAB3 at their protein (Figure 3D) but not mRNA levels (Additional file 4: Figure S4C), whereas the antagonism of endogenous miR-26b expression increased the protein levels of TAK1 and TAB3 (Figure 3E).

All together, these data imply that miR-26b may repress the expression of TAK1 and TAB3 by binding to their 3’UTR and thus blocking NF-κB signaling.

### miR-26b abrogates the doxorubicin-induced NF-κB activation and sensitizes HCC cells to the doxorubicin-induced apoptosis

**Figure 4:**
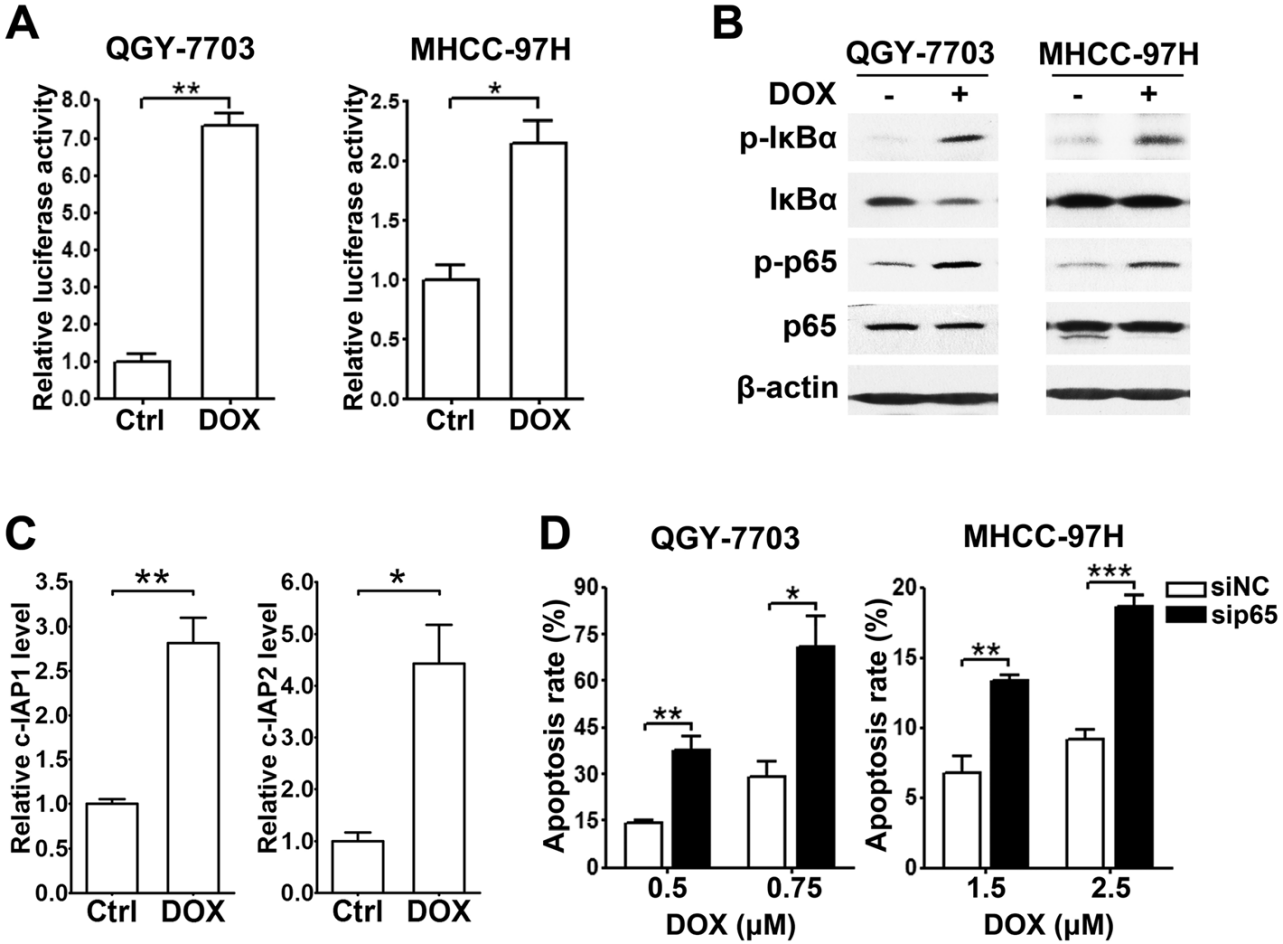
**Doxorubicin activates NF-κB signaling and knockdown of NF-κB promotes the doxorubicin-induced apoptosis. (A)** Doxorubicin enhanced NF-κB reporter activity. HCC cells transfected with pRL-TK and pNF-κB-Luc or pTAL-Luc were untreated (Ctrl) or treated (DOX) with doxorubicin for 12 hours before luciferase activity analysis. **(B)** Doxorubicin treatment increased the phosphorylation of IκBα and p65. HCC cells were untreated (-) or treated (+) with doxorubicin for 6 hours before immunoblotting. **(C)** Doxorubicin stimulated the expression of NF-κB target genes. QGY-7703 cells were untreated (Ctrl) or treated (DOX) with doxorubicin for 24 hours before qPCR analysis. **(D)** Knockdown of p65 sensitized HCC cells to the doxorubicin-triggered apoptosis. HCC cells transfected with siNC or sip65 duplex were treated with indicated concentrations of doxorubicin for 48 hours before the morphological analysis for apoptosis by DAPI staining. *, *P* < 0.05; **, *P* < 0.01; ***, *P* < 0.001.

Doxorubicin, an anthracycline commonly used in anti-cancer therapy, can trigger cell apoptosis by creating DNA double-strand breaks [21]. Doxorubicin is reported to promote the nuclear translocation and DNA-binding activity of NF-κB in HCC cells [22], but its biological consequence remains unknown. We found that doxorubicin treatment significantly enhanced the NF-κB reporter activity (Figure 4A), increased the levels of phosphorylated IκBα and p65 (Figure 4B), and induced the expression of *c-IAP1* and *c-IAP2* (Figure 4C). Importantly, compared with the negative control RNA duplex (siNC)-transfection, knockdown of p65 (Additional file 5: Figure S5) obviously increased the apoptosis rates in the doxorubicin-treated cells (Figure 4D). These data suggest that the doxorubicin-triggered NF-κB activation is protective against apoptosis, which may reduce the chemosensitivity of HCC cells.

**Figure 5:**
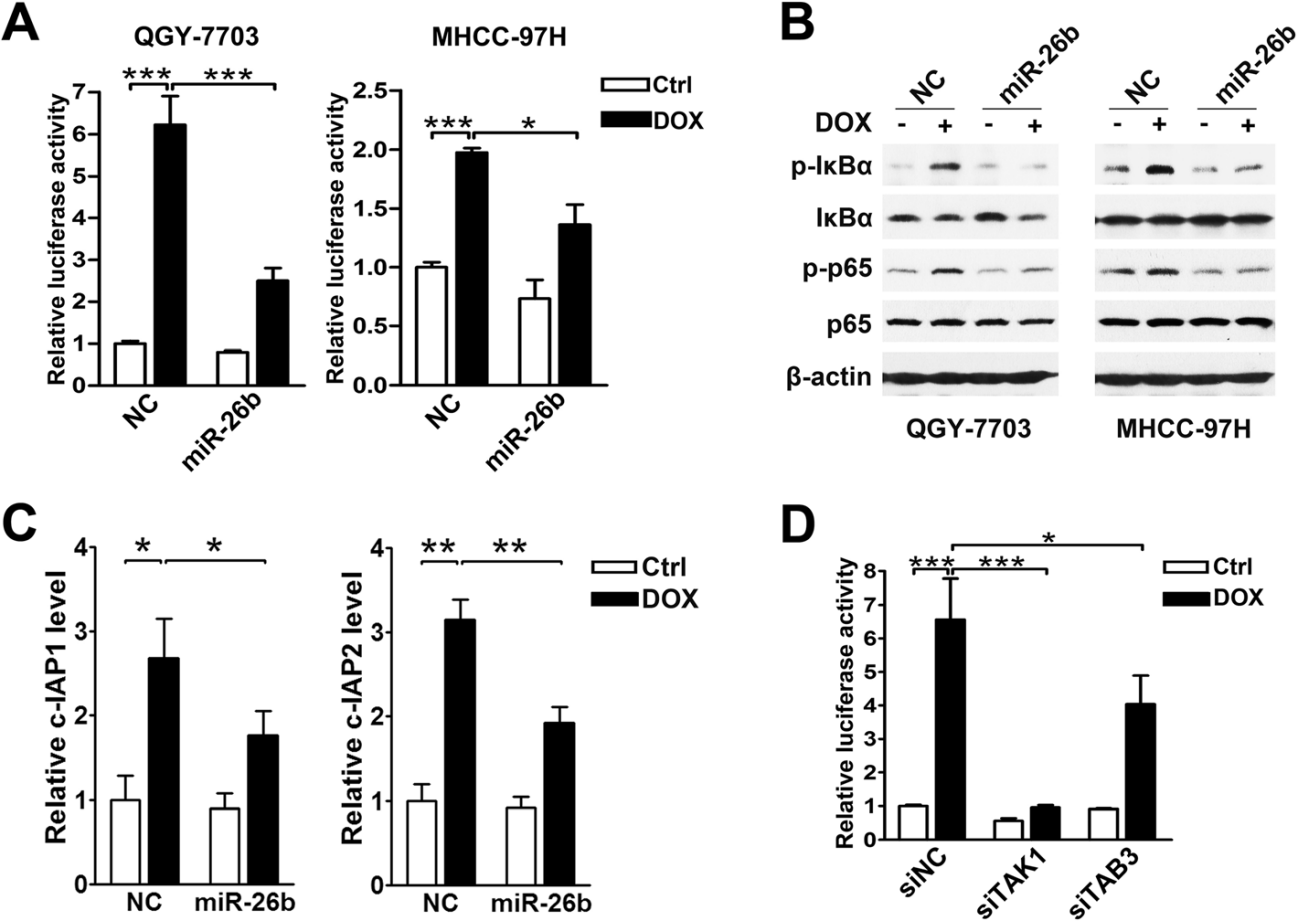
**miR-26b abrogates the doxorubicin-activated NF-κB signaling. (A)** Expression of miR-26b reduced the doxorubicin-induced NF-κB reporter activity. HCC cells were first transfected with NC or miR-26b duplexes, followed by co-transfection of pRL-TK and pNF-κB-Luc or pTAL-Luc, then remained untreated (Ctrl) or treated (DOX) with doxorubicin before luciferase activity analysis. **(B)** Introduction of miR-26b attenuated the doxorubicin-triggered phosphorylation of IκBα and p65. HCC cells transfected with NC or miR-26b were untreated (-) or treated (+) with doxorubicin for 6 hours before immunoblotting. **(C)** miR-26b suppressed the doxorubicin-induced expression of NF-κB target genes. QGY-7703 cells transfected with NC or miR-26b duplexes were untreated (Ctrl) or treated (DOX) with doxorubicin for 24 hours before qPCR analysis. **(D)** Knockdown of *TAK1* or *TAB3* suppressed the doxorubicin-stimulated NF-κB reporter activity. QGY-7703 cells were first transfected with the indicated siRNA duplexes, followed by treatment and analysis as in Figure 5A. *, *P* < 0.05; **, *P* < 0.01; ***, *P* < 0.001.

We then explored the effect of miR-26b on the doxorubicin-triggered NF-κB activation. The introduction of miR-26b significantly reduced the doxorubicin-induced NF-κB reporter activity, compared with NC transfection (Figure 5A). In addition, the doxorubicin-triggered phosphorylation of IκBα and p65 was profoundly attenuated in miR-26b-transfectants (Figure 5B). Furthermore, the ectopic expression of miR-26b effectively decreased the doxorubicin-stimulated expression of *c-IAP1* and *c-IAP2* (Figure 5C). Similar to the effect of miR-26b overexpression, knockdown of either *TAK1* or *TAB3* led to decreased NF-κB reporter activity in the doxorubicin-exposed cells (Figure 5D). These results suggest that miR-26b may inhibit the doxorubicin-induced NF-κB activation by targeting TAK1 and TAB3.

**Figure 6:**
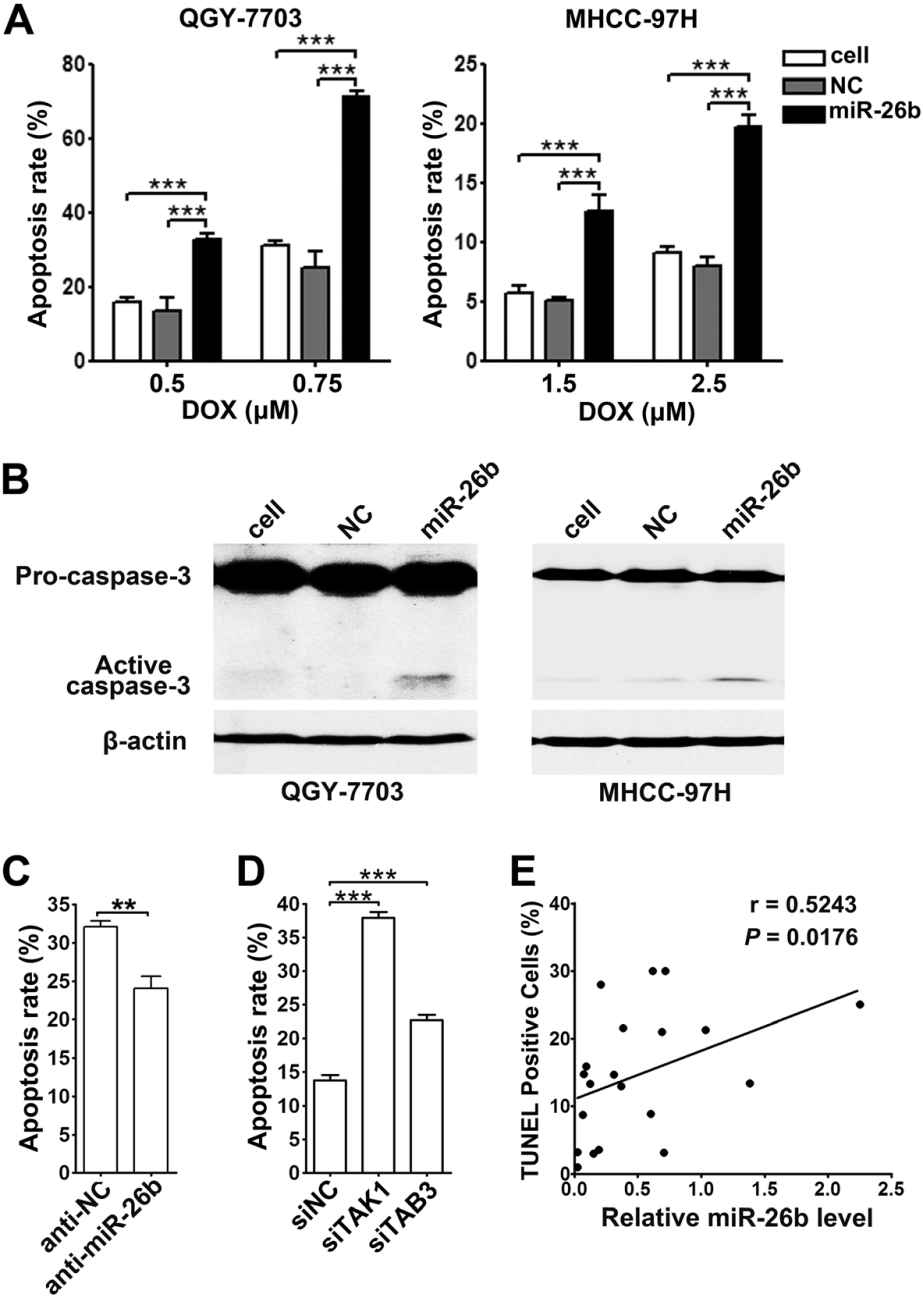
**miR-26b sensitizes tumor cells to the doxorubicin-induced apoptosis. (A)** Introduction of miR-26b sensitized HCC cells to the doxorubicin-induced apoptosis. Nontransfected (cell) or NC- or miR-26b-transfected HCC cells were treated with the indicated concentrations of doxorubicin (DOX) for 48 hours before apoptosis analysis by DAPI staining. **(B)** miR-26b expression increased the cleavage of pro-caspase-3. Nontransfected (cell) or NC- or miR-26b-transfected HCC cells were treated with doxorubicin for 48 hours before immunoblotting. **(C)** Antagonism of miR-26b desensitized tumor cells to the doxorubicin-induced apoptosis. QGY-7703 cells transfected with anti-NC or anti-miR-26b were treated with doxorubicin for 48 hours before apoptosis analysis by DAPI staining. **(D)** Knockdown of *TAK1* or *TAB3* sensitized HCC cells to the doxorubicin-induced apoptosis. QGY-7703 cells transfected with the indicated siRNA duplexes were treated with doxorubicin for 48 hours before apoptosis analysis by DAPI staining. **(E)** miR-26b expression is positively correlated with the rate of apoptosis in HCC tissues. The level of miR-26b was examined by real-time qPCR and normalized to RNU6B expression. Apoptosis was analyzed using TUNEL staining. The correlation between miR-26b levels and apoptosis rates in 20 HCC tissues was determined using Spearman’s correlation coefficient. **, *P* < 0.01; ***, *P* < 0.001.

The above observations disclosed that the doxorubicin-triggered NF-κB activation protected cells from apoptosis and miR-26b significantly inhibited NF-κB signaling in HCC cells, we therefore further analyzed whether miR-26b could sensitize tumor cells to the doxorubicin-induced apoptosis. Compared with NC- or non-transfected cells, the doxorubicin-induced apoptosis was much more pronounced in miR-26b-transfectants, as determined by the morphological examination with DAPI staining (Figure 6A). Moreover, immunoblotting assays revealed more active caspase-3 in the doxorubicin-exposed miR-26b-tranfectants than the control cells (Figure 6B). To verify the findings from gain-of-function study, loss-of-function analysis was performed. In response to doxorubicin treatment, anti-miR-26b-transfectants displayed reduced apoptosis rate, compared with the control group (Figure 6C). Similar to the phenotype induced by miR-26b expression, the silencing of either *TAK1* or *TAB3* enhanced the rates of doxorubicin-induced apoptosis (Figure 6D). These findings imply that miR-26b may inhibit the doxorubicin-triggered NF-κB signaling via targeting TAK1 and TAB3, which in turn sensitizes HCC cells to the doxorubicin-induced apoptosis.

We and others have previously shown that miR-26b is downregulated in HCC [15,16]. To confirm the association of miR-26b with the anti-apoptosis activity of NF-κB *in vivo*, we further evaluated the miR-26b levels and the apoptosis rates in human HCC specimens. Linear regression analysis disclosed a significant positive correlation between the level of miR-26b and the percentage of apoptotic cells in HCC tissues (Figure 6E and Additional file 6: Figure S6), suggesting that miR-26b downregulation may promote the survival of HCC cells *in vivo*.

## Discussion

NF-κB is frequently activated in the various types of tumors and promotes cancer development and chemoresistance. Therefore, miRNAs that possess the NF-κB inhibitory activity may provide novel targets for anti-cancer therapy.

The aberrant activation of NF-κB pathway may result from different causative mechanisms, like inactivating mutations or deletions of *IκBα* in Hodgkin’s lymphoma [23], activating mutations of *NF-κB2* gene in B- and T-cell lymphomas [23], amplifications and/or translocations of *NIK* in multiple myeloma [2]. In addition to the genomic aberrations of protein-coding genes, deregulation of the miRNAs that regulate the NF-κB pathway also results in the abnormal activation of the NF-κB pathway. For example, the NF-κB activators miR-301a and miR-30e* are overexpressed in pancreatic cancer and glioma, respectively [5,6]; the NF-κB inhibitor miR-195 is downregulated in HCC [9]. The decreased expression of the miR-26 family members has been observed in HCC tissues and the low miR-26a/b levels were associated with the short survival of patients [15,16]. Our data reveal a significant inhibitory role of miR-26b on NF-κB signaling, suggesting miR-26b downregulation as a novel mechanism that contributes to the abnormal activation of the NF-κB pathway in HCC cells.

Doxorubicin and its analogs, like daunorubicin, have gained broad application for the chemotherapy of various malignant tumors. They trigger the apoptosis of cancer cells by interfering with the actions of DNA topoisomerase IIα and creating DNA double-strand breaks [21]. Upon DNA damage, ataxia telangiectasia mutated (ATM) and sumoylated NF-κB essential modulator (NEMO) are jointly exported from the nucleus and mediates the TAK1/TAB2/3-dependent IKK activation [4]. Activated IKK then induces the phosphorylation and degradation of IκBα, which further liberates NF-κB to the nucleus and stimulates its DNA-binding activity. In lymphoma and cervical cancer cells, treatment with doxorubicin/daunorubicin enhances the transactivation activity of NF-κB [24,25]. On the other hand, although doxorubicin/daunorubicin also induces the phosphorylation and degradation of IκBα and increases the DNA-binding activity of NF-κB in osteosarcoma and breast cancer cells [26,27], yet they repress the NF-κB reporter activity and the expression of the NF-κB-regulated anti-apoptosis genes. Histone deacetylase has been shown to be recruited to p65 in the daunorubicin-treated osteosarcoma cells, which converts p65 into an active transcription repressor of anti-apoptosis genes [26]. In breast cancer cells, NF-κB induced by doxorubicin is deficient in phosphorylation and acetylation and represses the NF-κB-dependent transcription in a histone deacetylases-independent manner. The cellular context-dependent response calls for a cell-type-specific analysis in determining the outcome of doxorubicin-stimulated NF-κB signaling. Herein, we disclosed that doxorubicin markedly increased the level of phosphorylated p65 at the serine-536 residue and obviously activated the anti-apoptosis genes in HCC cells. Furthermore, the depletion of p65 dramatically increased the apoptosis rates of doxorubicin-exposed HCC cells, suggesting that NF-κB activation impairs the chemosensitivity of tumor cells and the inhibition of NF-κB signaling represents an effective strategy to overcome the chemoresistance of HCC to doxorubicin. Consistently, it has been shown that doxorubicin treatment activates NF-κB in other types of cancer cell lines, and blocking NF-κB activation sensitizes these cells to doxorubicin-triggered apoptosis [28]. Importantly, we found that the restoration of miR-26b expression significantly inhibited the phosphorylation of IκBα and p65, reduced the NF-κB reporter activity, blocked the nuclear translocation of NF-κB, consequently abrogated the expression of anti-apoptosis genes and sensitized HCC cells to the doxorubicin-induced apoptosis in HCC cells. These observations indicate miR-26b as a potent NF-κB inhibitor that may increase the chemosensitivity of HCC cells.

TAK1 is a member of the mitogen-activated protein kinase kinase kinase (MAP3K) family. It mediates the activation of IKK in various cell systems [3]. Upregulation of TAK1 is found in clear cell renal cell carcinomas and aggressive esophageal squamous cell carcinomas [29,30]. TAB3 is markedly overexpressed in skin, testis and small intestinal cancers [31]. Our results showed that miR-26b could decrease the protein level of TAK1 and TAB3 by directly binding to their 3’UTR. Considering that the expression of miR-26 family is frequently reduced in multiple types of cancer [15-19], we suggest that miR-26b downregulation may represent one of the mechanisms responsible for the overexpression of TAK1 and TAB3 in cancers.

In previous studies, miR-26a/b have been shown to block the G1/S transition of the cell cycle by targeting CCND2, CCNE1/2, CDK6, and EZH2 in HCC and nasopharyngeal carcinoma [15,18,32], and to restrain metastasis by suppressing the expression of IL6 in HCC [33]. miR-26a/b have also been reported to induce cell apoptosis by targeting MTDH, EZH2 and SLC7A11 in breast cancer cells and by targeting SODD in melanoma cells [17,19,34]. Our findings suggest that miR-26b may also suppress NF-κB signaling and enhance the chemosensitivity of hepatocellular carcinoma cells by targeting TAK1 and TAB3. It is exciting to find that a single miRNA may suppress tumor growth via multiple mechanisms, which makes miR-26b a promising anti-cancer target.

## Conclusions

In conclusion, this study demonstrated that miR-26b suppressed the TNFα- and doxorubicin-activated NF-κB signaling in HCC cells, and dramatically sensitized cancer cells to the doxorubicin-induced apoptosis. miR-26b exerted its inhibitory effect on the NF-κB pathway by repressing the expression of TAK1 and TAB3. Furthermore, the downregulation of miR-26b was correlated with the reduced apoptosis rate in HCC tissues. These findings highlight miR-26b as a potent inhibitor of the NF-κB pathway and an attractive target for cancer treatment.

## Methods

### Human tissue specimens

Histologically confirmed HCC tissues were collected from 20 patients who underwent HCC surgical resection at the Cancer Center of Sun Yat-sen University in Guangzhou, P.R. China. None of the patients had received any local or systemic anticancer treatment prior to the surgery. This study was approved by the Institute Research Ethics Committee at the Cancer Center and informed consent was obtained from each patient.

### Cell lines and transient transfection

Human hepatocellular carcinoma cell lines QGY-7703 and MHCC-97H were maintained in Dulbecco’s modified Eagle’s medium (DMEM, Invitrogen Corp., Buffalo, NY, USA) supplemented with 10% fetal bovine serum (FBS, Hyclone, Thermo Fisher Scientific, Victoria, Australia).

RNA oligos were reversely transfected using Lipofectamine RNAiMAX (Invitrogen, Carlsbad, CA, USA). A final concentration of 50 nM RNA duplex or 200 nM miRNA inhibitor was used. Transfection of plasmid DNA alone or together with RNA duplex was conducted using Lipofectamine 2000 (Invitrogen).

### RNA oligoribonucleotides

hsa-miR-26b (Pre-miR miRNA Precursor Product, cat. AM17100) and its negative control (Pre-miR Negative Control #2, cat. AM17111) were purchased from Applied Biosystems (Foster City, CA, USA). The sequence-specific miR-26b inhibitor (anti-miR-26b, cat. miR20000083) and its control (anti-NC, cat. miR02201) were obtained from Ribobio (Guangzhou, P.R. China). All siRNA duplexes were purchased from GenePharma (Shanghai, P.R. China). siTAK1, siTAB3 and sip65 targeted the mRNAs of human *TAK1* [GenBank: NM_145333], *TAB3* [GenBank: NM_152787] and *p65* [GenBank: NM_001145138] genes, respectively. The negative control RNA duplex (siNC) for siRNA was non-homologous to any human genome sequences. The sequences of all siRNA duplexes are listed in Additional file 7: Table S1.

### Vectors and luciferase reporter assay

To quantitatively examine NF-κB activity, luciferase reporter plasmid containing the minimal promoter with multiple tandem NF-κB binding sites (pNF-κB-Luc, Clontech, Palo Alto, CA, USA) and its control vector (pTAL-Luc, Clontech) were employed. Cells were first reversely transfected with 50 nM RNA duplex in a 48-well plate for 24 hours, followed by co-transfection with 10 ng pRL-TK (Promega, Madison, WI, USA) and 50 ng pNF-κB-Luc or pTAL-Luc for 32 hours, then remained untreated or treated with 20 ng/ml TNFα (cat. 210-TA-010, R&D Systems, Oxon, UK) for 4 hours or doxorubicin hydrochloride (cat. D1515, Sigma-Aldrich, St. Louis, MO, USA) for 12 hours before luciferase activity analysis.

To verify the miR-26b-targeted 3’UTR, firefly luciferase reporter plasmids pGL3cm-TAK1-3’UTR-WT, pGL3cm-TAK1-3’UTR-MUT, pGL3cm-TAB3-3’UTR-WT and pGL3cm-TAB3-3’UTR-MUT were constructed. The 3’UTR segment of human *TAK1* (529 bp) or *TAB3* (540 bp) mRNA that contained the putative wild-type (WT) or mutant (MUT) miR-26b binding site (Additional file 3: Figure S3) was PCR-amplified and inserted into the *Eco*RI and *Xba*I sites downstream of the stop codon of firefly luciferase in pGL3cm vector, which was created based upon the pGL3-control vector (Promega), as previously described [14]. The sequences of all primers are listed in Additional file 7: Table S1. Cells cultured in a 48-well plate were co-transfected with 20 nM RNA duplex, 10 ng pRL-TK and 20 ng firefly luciferase reporter containing the wild-type or mutant 3’UTR of target genes for 48 hours before luciferase activity analysis.

Luciferase activity was measured using the dual-luciferase reporter assay system (Promega). pRL-TK, which expresses *Renilla* luciferase, was used as an internal control to adjust for discrepancies in both transfection and harvest efficiencies. The luciferase activity of miR-26b-transfectants was normalized to the mean luciferase activity of NC-transfectants.

### Immunofluorescent staining for p65

Cells cultured on coverslips in a 48-well plate, were reversely transfected with 50 nM RNA duplexes for 48 hours and remained untreated or treated with 20 ng/mL TNFα for 10 minutes. Then the cells were fixed with 4% paraformaldehyde (PFA, cat. 16005, Sigma-Aldrich) and stained with rabbit monoclonal antibody (mAb) against p65 (cat. #4764, Cell Signaling Technology, CST, Beverly, MA, USA), followed by incubation with HiLyte Fluor 555-conjugated goat anti-rabbit IgG (cat. 28176-05-H555, AnaSpec, Fremont, CA, USA) and nuclear counterstaining with DAPI (cat. D9542, Sigma-Aldrich). Fluorescent pictures were photographed with Zeiss Axio Imager Z1 (Zeiss, Jena, Germany).

### RNA extraction and real-time quantitative RT-PCR

Total RNA was extracted from cultured cells using TriPure Isolation Reagent (cat. 11667165001, Roche Applied Science, Germany) according to the manufacturer’s instructions.

For real-time quantitative RT-PCR (qPCR) analysis of mRNA, 2 μg of total RNA was subjected to DNaseI digestion (Fermentas, Hanover, MD, USA) at 37°C for 30 minutes and then to heat inactivation of DNaseI at 65°C for 10 minutes, followed by reverse-transcription using Moloney murine leukemia virus reverse transcriptase (Promega). mRNA level was detected using Power SYBR® Green PCR Master Mix (Applied Biosystems) and *β-actin* was used as an internal control. The primers used for qPCR are listed in Additional file 7: Table S1. For qPCR analysis of miRNA, cDNA was synthesized using the Taqman miRNA reverse transcription kit (Applied Biosystems). The expression levels of miR-26b and the reference gene RNU6B were quantified using the TaqMan MicroRNA Assay Kit (Applied Biosystems).

All reactions were performed on a LightCycler® 480 (Roche Diagnostics, Germany) and were run in triplicate. The cycle threshold (Ct) values did not differ by more than 0.5 among the triplicates. The levels of target genes were normalized to the levels of the internal control genes to permit the calculation of the 2^-ΔΔCt^ value.

### Immunoblotting

Cellular proteins were separated in SDS-polyacrylamide gels, electrophoretically transferred to polyvinylidene difluoride membranes (cat. #162-0177, Bio-Rad, Cambridge, MA, USA), then detected with antibodies. The sources of antibodies were as follows: mouse mAb against IκBα (cat. #4814, CST), phospho-Ser32/Ser36 of IκBα (cat. 551818, BD, Franklin Lakes, NJ, USA) and β-actin (cat. BM0627, Boster, Wuhan, China); rabbit mAb for p65 (cat. #4764, CST), phospho-Ser536 of p65 (cat. #3033, CST) and TAK1 (cat. #5206, CST); rabbit polyclonal antibodies for TAB3 (cat. ab85655, Abcam, Cambridge, MA, USA) and caspase-3 (cat. #9662, CST). β-actin was used as an internal control. All results were reproduced in three independent experiments, and the representative immunoblots are shown.

### Apoptosis analysis

For cultured cells, 24 hours after transfection, cells were treated with doxorubicin for 48 hours, then applied to morphological examination and detection of caspase-3 activity. For morphological examination, the cells were fixed with 4% PFA, stained with DAPI and those with condensed or fragmented nuclei were considered as apoptotic cells. At least 500 cells were counted for each sample. The activity of caspase-3 was detected by immunoblotting. Activated caspase-3 resulting from the cleavage of the inactive proenzyme form was indicated as 17/19 kDa bands below the full length caspase-3 (35 kDa) band.

For HCC tissues, TUNEL staining was performed using the In Situ Cell Death Detection Kit (cat. 11684817910, Roche Applied Science), according to the manufacturer’s protocol. At least 750 cells were counted for each sample.

### Statistical analysis

Data were expressed as the mean ± standard error of the mean (SEM) from at least three independent experiments. Analyses on the differences between groups were performed using GraphPad Prism version 4.0 (GraphPad Software, Inc., San Diego, CA, USA). Student’s *t* test was performed to compare the differences between two groups and one-way ANOVA was applied to compare more than two groups. Correlation between the miR-26b level and the apoptosis rate in HCC tissues was explored using Spearman’s correlation coefficient. All statistical tests were two-sided and *P* < 0.05 was considered to be statistically significant.

## Competing interests

The authors declare that they have no competing interests.

## Authors’ contributions

NZ and RW designed and performed experiments, discussed and interpreted the data. LZ and JG performed experiments. YZ gave suggestion on study design, discussed and interpreted the data. SMZ designed and supervised study, discussed and interpreted the data, wrote the manuscript. All authors read and approved the final manuscript.

## Supplementary Material

Additional file 1: Figure S1 miR-26b suppresses the TNFα-stimulated NF-κB signaling in MHCC-97H cells. **(A)** miR-26b inhibited the TNFα-induced nuclear translocation of NF-κB. Cells transfected with NC or miR-26b were untreated (Ctrl) or treated with TNFα before immunofluorescent staining for p65 (red). The nuclei were stained blue with DAPI. Scale bar, 20 μm. **(B)** miR-26b suppressed the TNFα-induced expression of NF-κB target genes. Cells transfected with NC or miR-26b were treated with 20 ng/ml TNFα for 3 hours before qPCR analysis. *, *P* < 0.05; **, *P* < 0.01. Click here for file

Additional file 2: Figure S2 Reduction of endogenous miR-26b level by anti-miR-26b. QGY-7703 cells were transfected with anti-NC or anti-miR-26b for 48 hours before qPCR analysis. *, *P* < 0.05. Click here for file

Additional file 3: Figure S3 miR-26b and its putative binding sequences in the 3’UTRs of *TAK1* and *TAB3*. The mutant miR-26b-binding sites (underlined) were generated in the complementary sites for the seed region of miR-26b. Click here for file

Additional file 4: Figure S4 Effects of siRNA and miR-26b on the expression of cellular TAK1 and TAB3 in QGY-7703 cells. **(A-B)** siTAK1 and siTAB3 silenced the expression of TAK1 and TAB3 proteins. Cells were transfected with siNC or siRNA targeting *TAK1***(A)** or *TAB3***(B)** for 48 hours before immunoblotting. **(C)** miR-26b had no effect on the mRNA level of *TAK1* and *TAB3*. Cells were transfected with NC or miR-26b duplexes for 48 hours before qPCR analysis. Click here for file

Additional file 5: Figure S5 Knockdown of endogenous p65 protein by siRNA. HCC cells were transfected with siNC or sip65 duplexes for 48 hours before immunoblotting. Click here for file

Additional file 6: Figure S6 Representative images of TUNEL staining in HCC tissues. Apoptotic cells exhibited brown staining. Scale bar, 50 μm. Click here for file

Additional file 7: Table S1 Sequences of RNA and DNA Oligonucleotides. Click here for file
