# Supplementary figures and images for: MicroRNA-26b suppresses the NF-κB signaling and enhances the chemosensitivity of hepatocellular carcinoma cells by targeting TAK1 and TAB3
Source: Mol Cancer. 2014 Feb 24;13:35. doi: 10.1186/1476-4598-13-35 (PMC3938074; doi:10.1186/1476-4598-13-35)

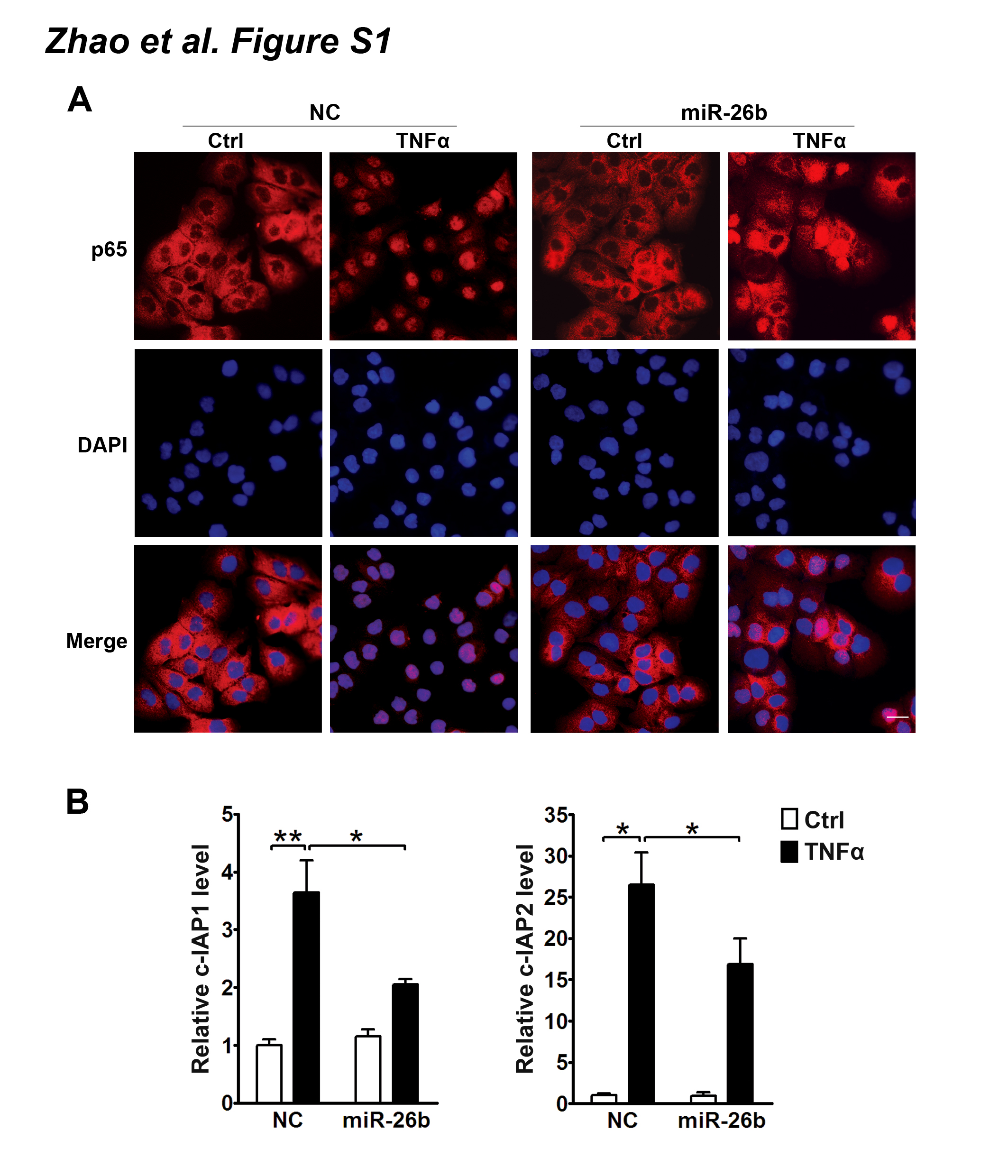

Supplement: Additional file 1: Figure S1 — miR-26b suppresses the TNFα-stimulated NF-κB signaling in MHCC-97H cells. (A) miR-26b inhibited the TNFα-induced nuclear translocation of NF-κB. Cells transfected with NC or miR-26b were untreated (Ctrl) or treated with TNFα before immunofluorescent staining for p65 (red). The nuclei were stained blue with DAPI. Scale bar, 20 μm. (B) miR-26b suppressed the TNFα-induced expression of NF-κB target genes. Cells transfected with NC or miR-26b were treated with 20 ng/ml TNFα for 3 hours before qPCR analysis. *, P < 0.05; **, P < 0.01. [file 1476-4598-13-35-S1.jpeg]

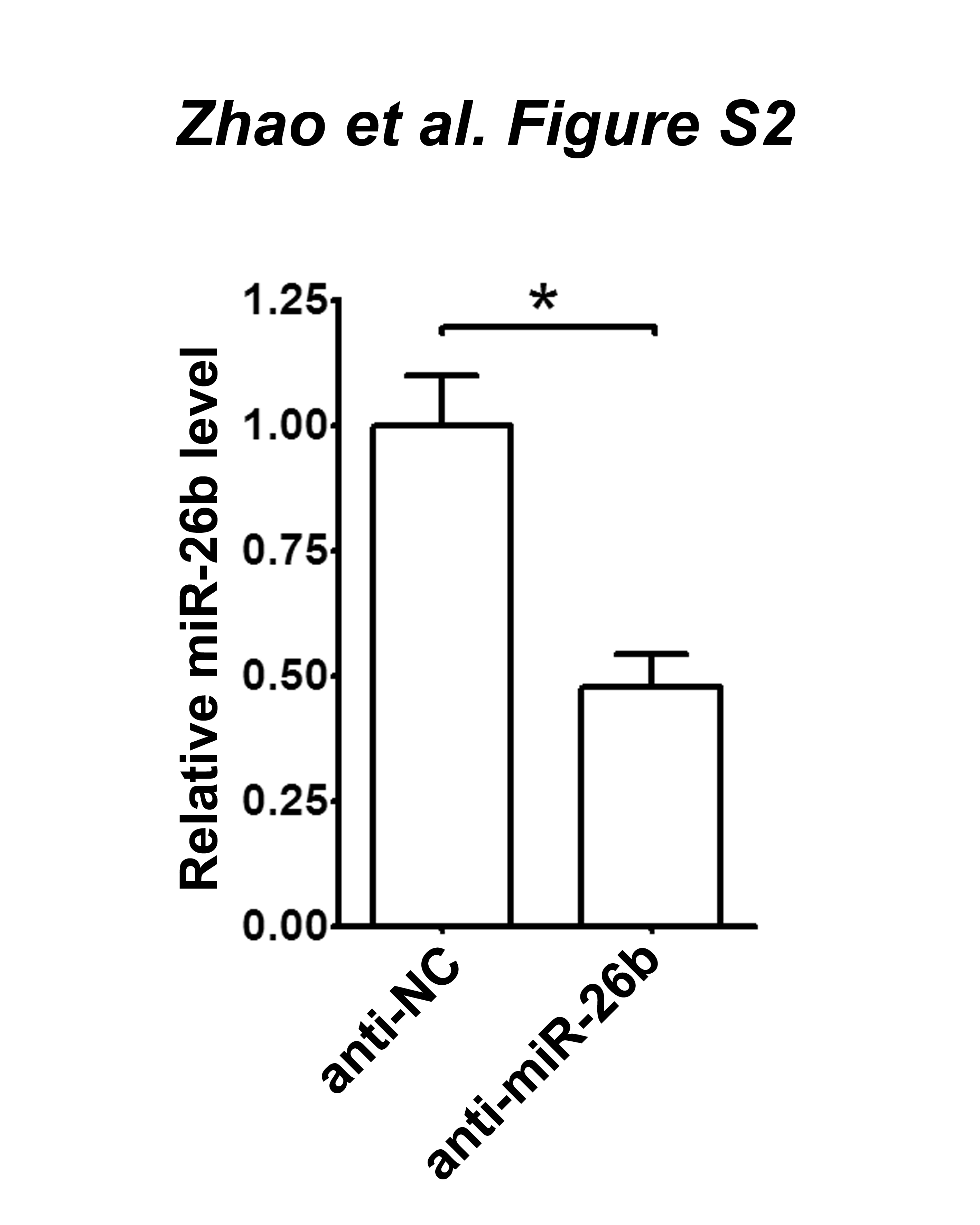

Supplement: Additional file 2: Figure S2 — Reduction of endogenous miR-26b level by anti-miR-26b. QGY-7703 cells were transfected with anti-NC or anti-miR-26b for 48 hours before qPCR analysis. *, P < 0.05. [file 1476-4598-13-35-S2.jpeg]

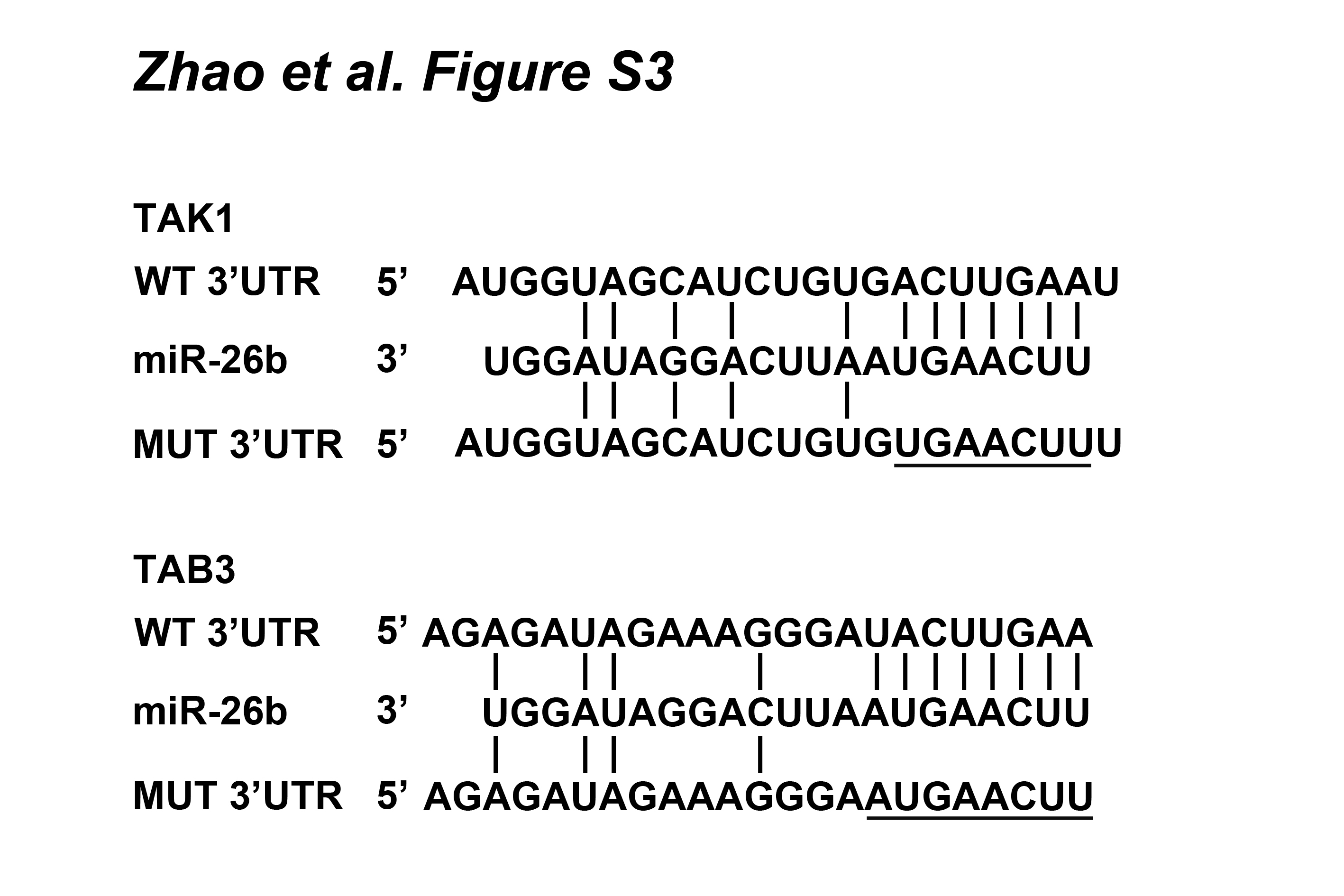

Supplement: Additional file 3: Figure S3 — miR-26b and its putative binding sequences in the 3’UTRs of TAK1 and TAB3. The mutant miR-26b-binding sites (underlined) were generated in the complementary sites for the seed region of miR-26b. [file 1476-4598-13-35-S3.jpeg]

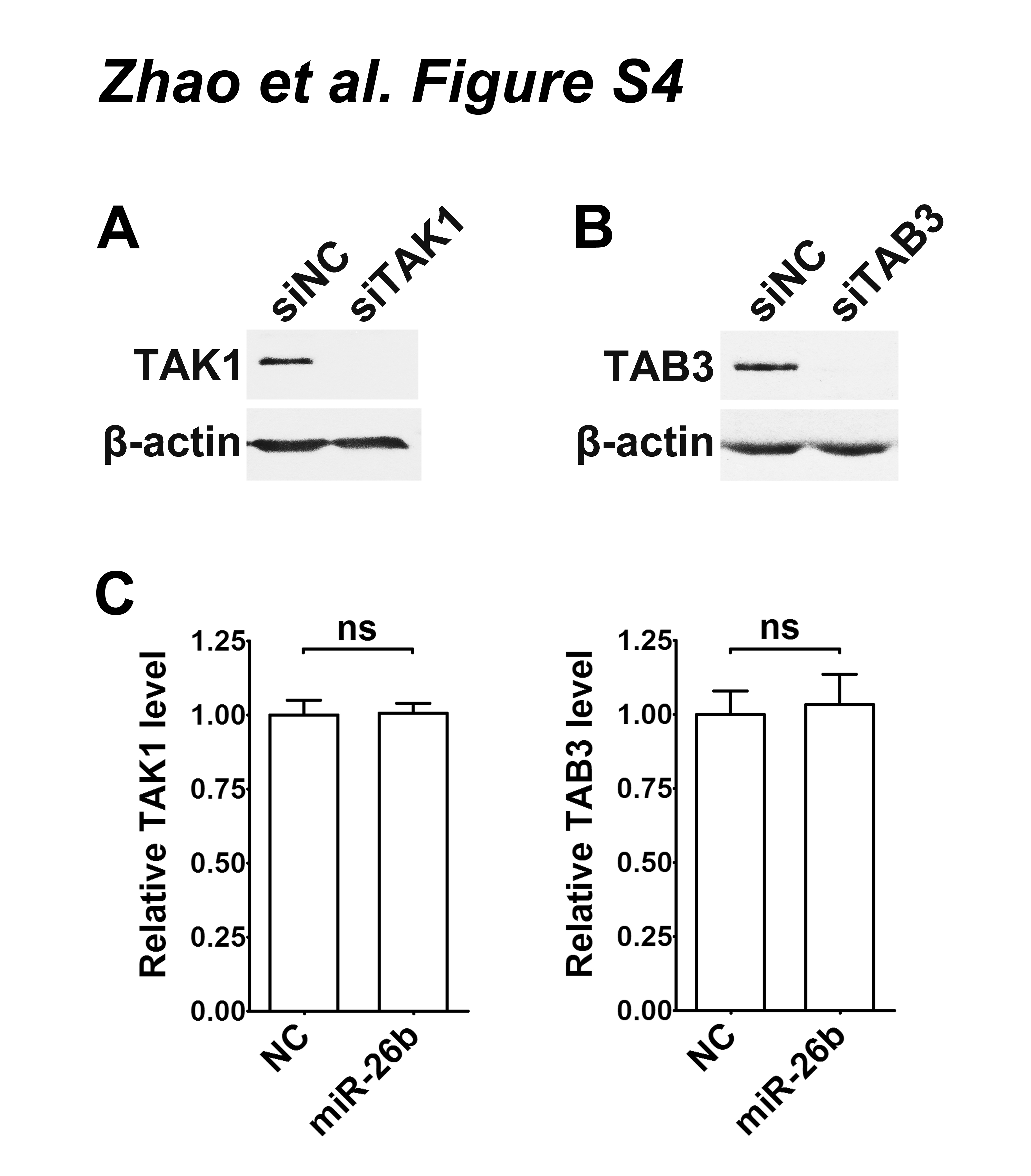

Supplement: Additional file 4: Figure S4 — Effects of siRNA and miR-26b on the expression of cellular TAK1 and TAB3 in QGY-7703 cells. (A-B) siTAK1 and siTAB3 silenced the expression of TAK1 and TAB3 proteins. Cells were transfected with siNC or siRNA targeting TAK1(A) or TAB3(B) for 48 hours before immunoblotting. (C) miR-26b had no effect on the mRNA level of TAK1 and TAB3. Cells were transfected with NC or miR-26b duplexes for 48 hours before qPCR analysis. [file 1476-4598-13-35-S4.jpeg]

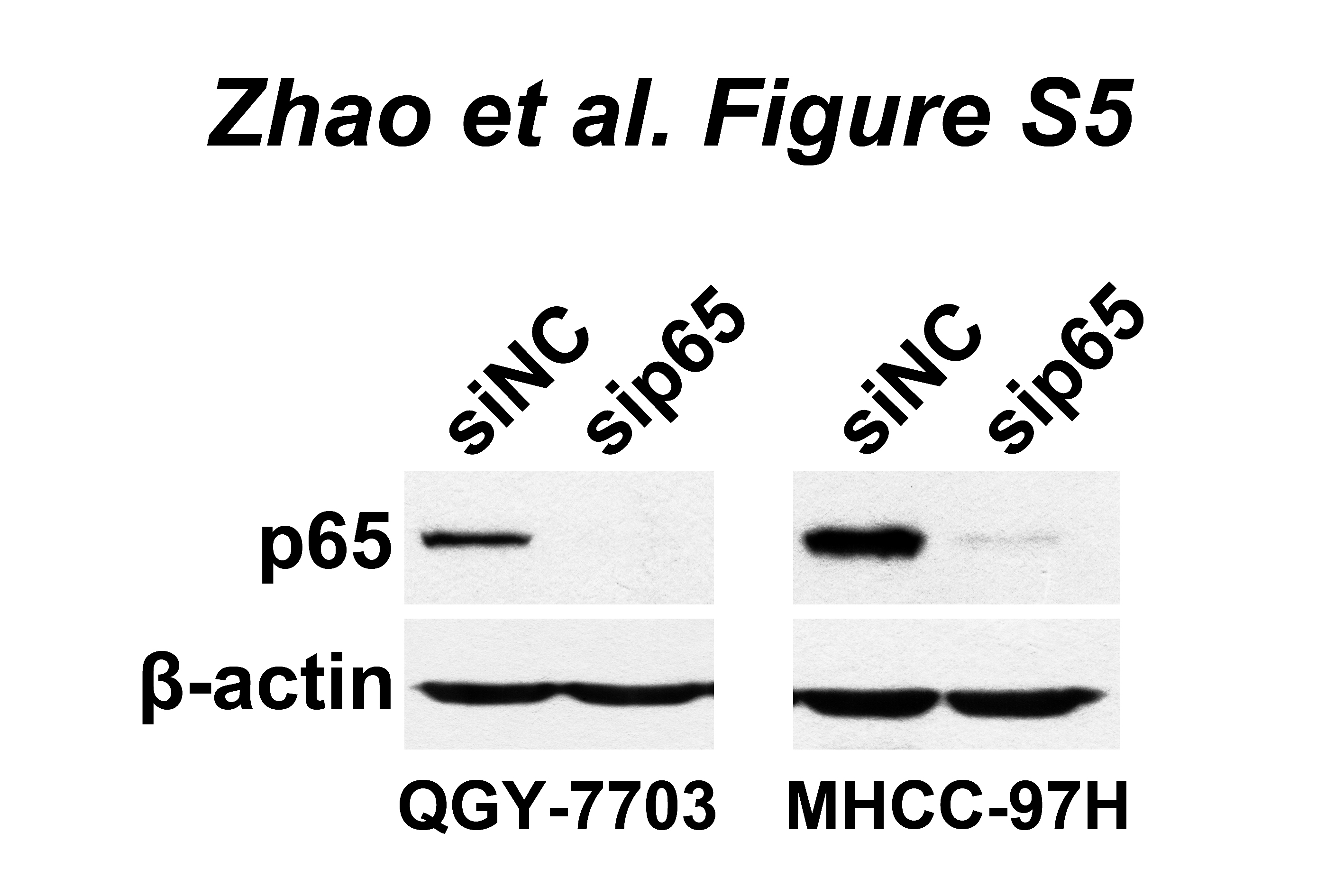

Supplement: Additional file 5: Figure S5 — Knockdown of endogenous p65 protein by siRNA. HCC cells were transfected with siNC or sip65 duplexes for 48 hours before immunoblotting. [file 1476-4598-13-35-S5.jpeg]

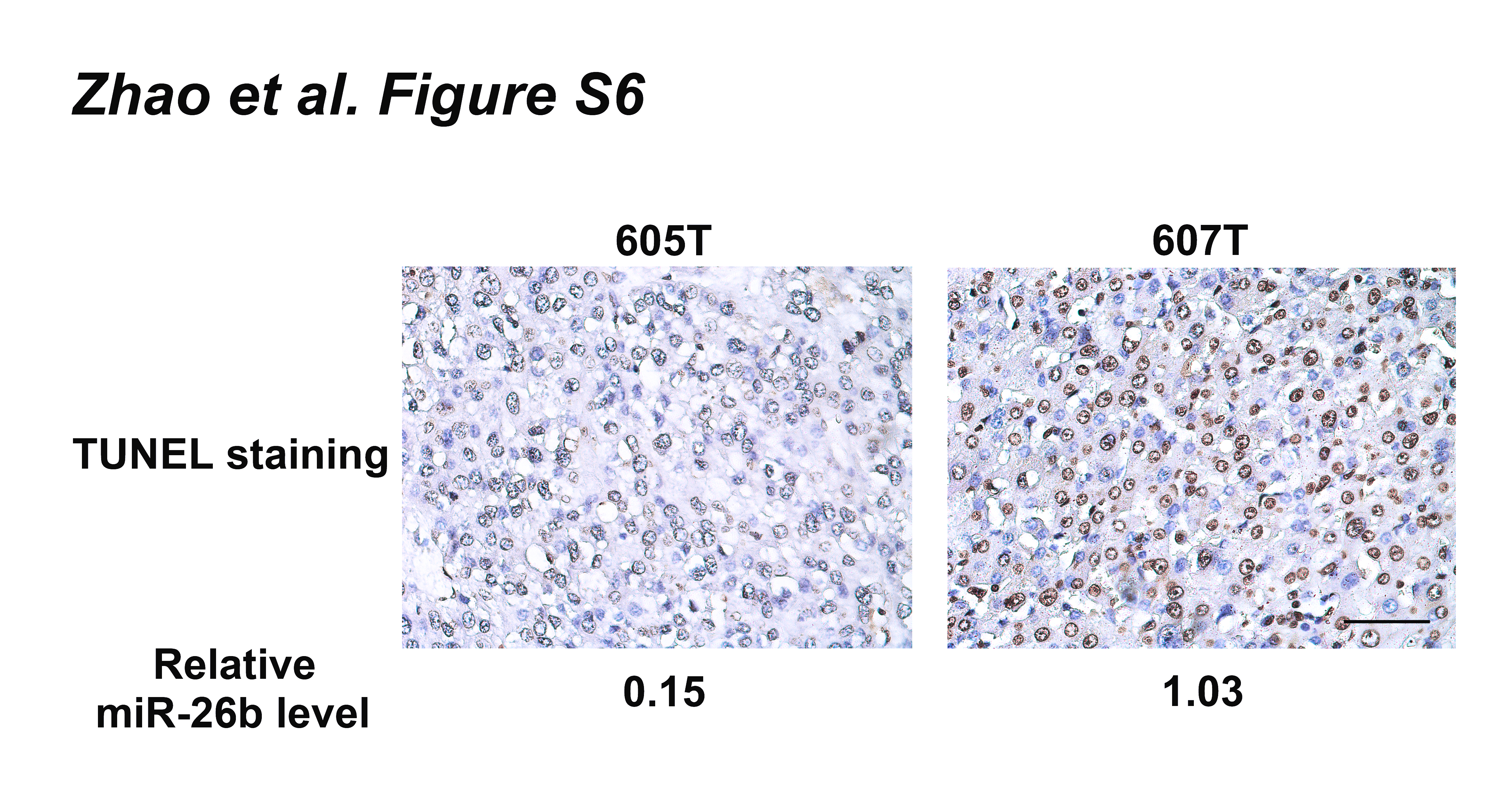

Supplement: Additional file 6: Figure S6 — Representative images of TUNEL staining in HCC tissues. Apoptotic cells exhibited brown staining. Scale bar, 50 μm. [file 1476-4598-13-35-S6.jpeg]
