# Supplementary material for: MicroRNA-26b suppresses the NF-κB signaling and enhances the chemosensitivity of hepatocellular carcinoma cells by targeting TAK1 and TAB3
Source: Mol Cancer. 2014 Feb 24;13:35. doi: 10.1186/1476-4598-13-35 (PMC3938074; doi:10.1186/1476-4598-13-35)
Supplement: Additional file 7: Table S1 — Sequences of RNA and DNA Oligonucleotides. [file 1476-4598-13-35-S7.doc]

**Table S**1. Sequences of RNA and DNA Oligonucleotides

| **Name** | **Sense Strand/Sense Primer (5'-3')** | **Antisense Strand/Antisense Primer (5'-3')** |
| --- | --- | --- |
| **siRNA Duplexes** | | |
| siTAK1 | GGUAGUAAUUACAGUGAAAdTdT | UUUCACUGUAAUUACUACCdTdT |
| siTAB3 | GAGCAAGGAUGGAGAGGUUdTdT | AACCUCUCCAUCCUUGCUCdGdT |
| sip65 | UGGAGUACCCUGAGGCUAUdTdT | AUAGCCUCAGGGUACUCCAUC |
| NC | UUGUACUACACAAAAGUACUG | GUACUUUUGUGUAGUACAGUU |
|  | | |
| **Primers for 3'UTR Cloning (Restriction enzyme sites were underlined)** | | |
| TAK1 3'UTR | ACGGAATTCctgggaccgttacattttga | ACGTCTAGAgcccttacacggaactatcc |
| TAB3 3'UTR | AGTGGGCCCtgaaactttgagcactaccaga | ACGTCTAGAtgttggaaattacccctgaa |
|  | | |
| **Primers for** **real-time quantitative RT-PCR** | | |
| c-IAP1 | gtcgcaatgatgatgtcaaa | ctcttggcctttcattcgta |
| c-IAP2 | caaatgcttttgctgtgatg | gggctgtctgatgtggatag |
| β-actin | ACTGGAACGGTGAAGGTGAC | AGAGAAGTGGGGTGGCTTTT |
| TAK1 | AGAGGAGCCTTTGGAGTTGT | CCATCACAAGACACACTGGA |
| TAB3 | GCCCATTTCAGTGATACCAG | CCGCTCTAGCTCCTCTTTCT |
